# Supplementary material for: Cold adaptation recorded in tree rings highlights risks associated with climate change and assisted migration
Source: Nat Commun. 2018 Apr 23;9:1574. doi: 10.1038/s41467-018-04039-5 (PMC5913219; doi:10.1038/s41467-018-04039-5)
Supplement: Supplementary file 1 — Supplementary Information [file 41467_2018_4039_MOESM1_ESM.pdf]

Supplementary Information

Cold adaptation recorded in tree rings highlights risks associated with climate change and assisted migration

Montwé *et al*.

Supplementary Table 1. Climate of provenance origin

Region	Prov.	Lat.	Long.	Elev.	MAT	MWMT	MCMT	TD	MAP	MSP	AHM	SHM	CMD
N	33	63.30	-136.47	876	-4.3	13.8	-24.6	38.4	423	249	14	55	131
N	30	59.98	-128.55	640	-2.7	14.5	-23.5	38.0	449	236	16	62	197
N	35	59.80	-133.78	789	-0.4	12.6	-15.0	27.6	361	186	27	68	203
N	28	58.67	-124.17	762	-1.3	13.4	-15.4	28.8	689	491	13	27	7
N	66	58.65	-124.77	1173	-1.7	10.8	-13.6	24.4	675	482	12	22	0
CI	100	55.80	-124.82	762	1.3	13.6	-12.0	25.6	522	251	22	54	181
CI	20	54.13	-127.23	937	1.8	12.7	-10.4	23.1	551	243	21	52	208
CI	104	54.02	-124.53	732	2.7	14.3	-10.9	25.2	506	236	25	61	267
CI	61	53.87	-121.80	838	2.7	14.1	-10.1	24.2	750	331	17	43	155
CI	107	52.50	-125.80	1311	1.0	10.7	-9.4	20.1	822	209	13	51	211
SI	14	50.97	-120.33	1059	3.9	15.0	-7.6	22.6	466	221	30	68	304
SI	72	50.70	-119.18	777	5.7	17.1	-6.3	23.4	735	312	21	55	256
SI	57	49.90	-118.20	579	5.9	17.4	-5.8	23.2	659	258	24	67	329
SI	1	49.58	-119.02	1006	4.6	15.9	-6.5	22.4	547	226	27	70	319
SI	42	49.18	-117.58	998	5.3	16.9	-6.1	23.0	858	285	18	59	265
US	111	47.78	-120.93	762	7.4	17.3	-1.5	18.8	1535	237	11	73	261
US	144	46.67	-113.67	1524	4.6	16.0	-5.7	21.7	408	189	36	85	426
US	153	45.63	-117.27	1311	5.7	16.2	-3.9	20.1	510	217	31	75	439
US	154	44.53	-118.57	1494	6.5	17.1	-2.6	19.7	676	167	24	102	491
US	123	44.38	-121.67	1006	7.4	16.8	-1.0	17.8	427	73	41	230	692

Climate variables are averaged across the 1961-1990 normal period. *Region* refers to the climatic region to which each provenance is assigned, where: North is abbreviated to “N”; Central Interior is shortened to “CI”; Southern Interior is abbreviated to “SI”; and “US” indicates seed sources from the United States of America. *Prov* refers to the number assigned to each seed source from the Illingworth provenance trial. Latitude (Lat.) and longitude (Long.) are provided in decimal degrees, while elevation (Elev.) is provided in meters above sea level. Temperature variables include: mean annual temperature (MAT, °C), mean warmest month temperature (MWMT, °C), mean coldest month temperature (MCMT, °C), and temperature difference (TD, °C). Precipitation variables include: mean annual precipitation (MAP, mm), and mean summer precipitation (MSP, mm). Moisture variables include: annual heat-moisture index (AHM), summer heat-moisture index (SHM), and climate moisture deficit is given in mm (CMD).

Supplementary Table 2. Climate of the three planting sites

Site	Lat.	Long.	Elev.	MAT	MWMT	MCMT	TD	MAP	MSP	AHM	SHM	CMD
CHUW	50.58	-120.62	1430	3.1	14	-7.1	21.1	482	241	27.2	58.1	232
COMM	50.92	-120.07	1370	3.1	13.9	-7.5	21.4	522	256	25.1	54.3	217
EQUI	50.37	-119.6	1370	3.3	14.2	-7.3	21.5	686	301	19.4	47.2	188

All climate variables are averaged across the 1961-1990 normal period. Site refers to the abbreviated site name from the Illingworth lodgepole pine provenance trial: CHUW is the abbreviation for Chuwels Lake, COMM stands for Community Lake, and EQUI stands for Equises Creek. Latitude (Lat.) and longitude (Long.) are provided in decimal degrees, while elevation (Elev.) is provided in meters above sea level. Temperature variables include: mean annual temperature (MAT, °C), mean warmest month temperature (MWMT, °C), mean coldest month temperature (MCMT, °C), and temperature difference (TD, °C). Precipitation variables include: mean annual precipitation (MAP, mm), mean summer precipitation (MSP, mm). Moisture variables include: annual heat-moisture index (AHM); summer heat-moisture index (SHM); and climate moisture deficit is given in mm (CMD).

Supplementary Table 3. Mixed model summary for blue ring intensity with population as fixed effect. The threshold coefficients refer to the ordinal intensity scores, ranging from 0 (absence) to 5 (highest intensity). DoY refers to the Julian day of year.

Response: Blue ring intensity

Coefficients:	Estimate	Std. Error	z value	p-value
Population North	-2.14	0.269	-7.96	< 0.001
Population Southern Interior	0.73	0.208	3.50	< 0.001
Population United States	0.44	0.212	2.07	0.039
Start of growing season (DoY)	0.29	0.052	5.50	< 0.001
End of growing season (DoY)	-0.25	0.066	-3.85	< 0.001
Annual sum of growing degree days over 5°C	-1.32	0.082	-16.11	< 0.001
Threshold coefficients:	Estimate	Std. Error	z value	
0|1	1.79	0.274	6.54	
1|2	2.67	0.278	9.62	
2|3	3.26	0.282	11.59	
3|4	4.16	0.291	14.33	
4|5	4.94	0.305	16.21	

Supplementary Table 4. Mixed model summary for blue ring intensity with diameter as fixed effects. The threshold coefficients refer to the ordinal intensity scores, ranging from 0 (absence) to 5 (highest intensity). DoY refers to the Julian day of year.

Response: Blue ring intensity

Coefficients:	Estimate	Std. Error	z value	p-value
Diameter	-1.47	0.070	-20.97	< 0.001
Start of growing season (DoY)	0.03	0.056	0.54	0.589
End of growing season (DoY)	-0.29	0.071	-4.14	< 0.001
Annual sum of growing degree days over 5°C	-1.50	0.102	-14.75	< 0.001
Threshold coefficients:	Estimate	Std. Error	z value	
0|1	2.51	0.487	5.17	
1|2	3.63	0.492	7.39	
2|3	4.42	0.496	8.91	
3|4	5.62	0.506	11.10	
4|5	6.57	0.517	12.71	

Supplementary Table 5. Mixed model summary for frost ring intensity at position 1 with population as fixed effect. The threshold coefficients refer to the ordinal intensity scores, ranging from 0 (absence) to 5 (highest intensity). DoY refers to the Julian day of year.

Response: Frost ring intensity at position 1

Coefficients	Estimate	Std. Error	z value	p-value
Population North	-0.05	0.238	-0.20	0.844
Population Southern Interior	0.70	0.215	3.28	0.001
Population United States	0.36	0.230	1.56	0.119
End of growing season (DoY) previous year	-0.43	0.107	-4.02	< 0.001
Annual sum of growing degree days over 5°C previous year	-0.95	0.123	-7.75	< 0.001
Threshold coefficients:	Estimate	Std. Error	z value	
0|1	3.40	0.229	14.82	
1|2	3.84	0.236	16.27	
2|3	4.20	0.244	17.26	
3|4	4.69	0.257	18.27	
4|5	5.44	0.289	18.82	

Supplementary Table 6. Mixed model summary for frost ring intensity at position 1 with diameter as fixed effects. The threshold coefficients refer to the ordinal intensity scores, ranging from 0 (absence) to 5 (highest intensity). DoY refers to the Julian day of year.

Response: Frost ring intensity at position 1

Coefficients:	Estimate	Std. Error	z value	p-value
Diameter	-2.60	0.195	-13.30	< 0.001
End of growing season (DoY) previous year	-0.57	0.116	-4.93	< 0.001
Annual sum of growing degree days over 5°C previous year	-0.65	0.172	-3.76	< 0.001
Threshold coefficients:	Estimate	Std. Error	z value	
0|1	4.89	0.300	16.30	
1|2	5.47	0.313	17.47	
2|3	5.93	0.325	18.23	
3|4	6.53	0.344	18.99	
4|5	7.39	0.377	19.61	

Supplementary Table 7. Mixed model summary for frost ring intensity at position 2. The threshold coefficients refer to the ordinal intensity scores, ranging from 0 (absence) to 5 (highest intensity). DoY refers to the Julian day of year.

Response: Frost ring intensity at position 2

Coefficients	Estimate	Std. Error	z value	p-value
Population North	0.10	0.294	0.34	0.731
Population Southern Interior	-0.49	0.331	-1.48	0.138
Population United States	-0.94	0.384	-2.45	0.014
Start of growing season (DoY)	0.54	0.099	5.46	< 0.001
Day of year at which the sum of growing degree days over 5°C has reached 100	-0.66	0.123	-5.35	< 0.001
Threshold coefficients:	Estimate	Std. Error	z value	
0|1	3.62	0.432	8.38	
1|2	4.57	0.452	10.11	
2|3	5.15	0.475	10.85	
3|4	5.73	0.512	11.19	
4|5	8.24	1.086	7.58	

Supplementary Table 8. Mixed model summary for frost ring intensity at position 2 with diameter as fixed effects. The threshold coefficients refer to the ordinal intensity scores, ranging from 0 (absence) to 5 (highest intensity). DoY refers to the Julian day of year.

Response: Frost ring intensity at position 2

Coefficients:	Estimate	Std. Error	z value	p-value
Diameter	-1.11	0.158	-7.05	< 0.001
Start of growing season (DoY)	0.36	0.100	3.58	< 0.001
Day of year at which the sum of growing degree days over 5°C has reached 100	-0.80	0.129	-6.16	< 0.001
Threshold coefficients:	Estimate	Std. Error	z value	
0|1	4.17	0.334	12.51	
1|2	5.14	0.361	14.24	
2|3	5.74	0.391	14.69	
3|4	6.32	0.436	14.51	
4|5	8.84	1.053	8.40	

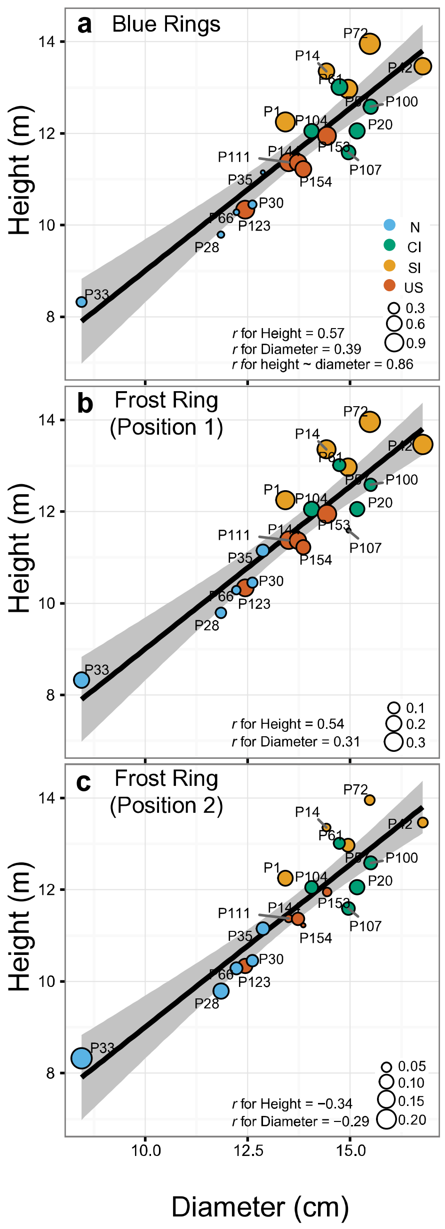

Supplementary Figure 1. Cold-growth trade-off. In all panels, the y-axis represents tree height in 2005 and the x-axis represents diameter at breast height (1.3 m) in 2005. All data points represent provenance response averaged from 6 replicates and are colored by climatic region. The size of the points corresponds to cold indicators: Panel a) displays blue ring intensities; panel b) illustrates frost ring intensities in position 1; and panel c) represents frost ring intensities in position 2. Spearman correlation coefficients are found in the lower right corner of each panel. These correlations are between height or diameter and, depending on the panel, one of the three measures of cold (blue ring intensity, frost ring in position 1, frost ring in position 2). The circles represent the provenances used in this study, colored by climatic region. These 20 provenances were grown at three planting sites in British Columbia's southern interior.

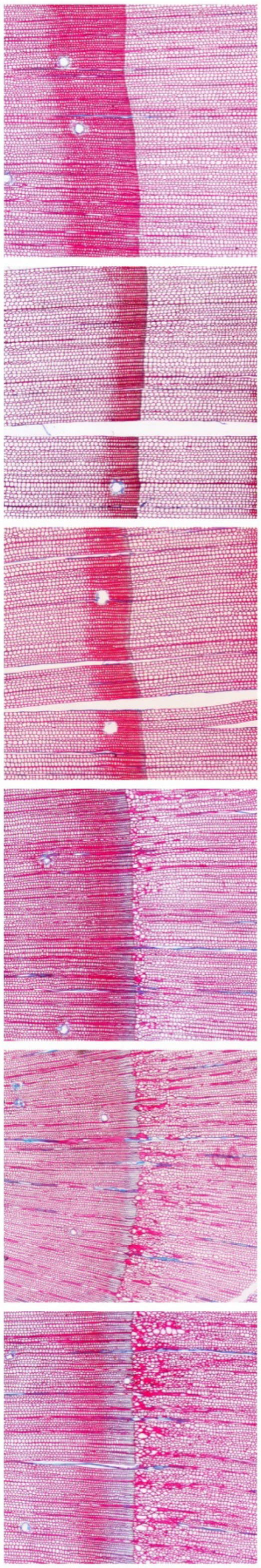

Supplementary Figure 2. Blue ring intensity classification scheme. A ranking of 0 indicates absence, a ranking of 1 represents at least one row of unlignified cells, and the strongest ranking (5) is associated with multiple rows of unlignified cells that are often associated with frost damage in position 1 in the next year's earlywood.
